# Late Byzantine Mineral Soda High Alumina Glasses from Asia Minor: A New Primary Glass Production Group

**DOI:** 10.1371/journal.pone.0018970

**Published:** 2011-04-19

**Authors:** Nadine Schibille

**Affiliations:** Research Laboratory for Archaeology and the History of Art, University of Oxford, Oxford, United Kingdom; Joint Research Centre - European Commission, Germany

## Abstract

The chemical characterisation of archaeological glass allows the discrimination between different glass groups and the identification of raw materials and technological traditions of their production. Several lines of evidence point towards the large-scale production of first millennium CE glass in a limited number of glass making factories from a mixture of Egyptian mineral soda and a locally available silica source. Fundamental changes in the manufacturing processes occurred from the eight/ninth century CE onwards, when Egyptian mineral soda was gradually replaced by soda-rich plant ash in Egypt as well as the Islamic Middle East. In order to elucidate the supply and consumption of glass during this transitional period, 31 glass samples from the assemblage found at Pergamon (Turkey) that date to the fourth to fourteenth centuries CE were analysed by electron microprobe analysis (EPMA) and by laser ablation-inductively coupled plasma-mass spectrometry (LA-ICP-MS). The statistical evaluation of the data revealed that the Byzantine glasses from Pergamon represent at least three different glass production technologies, one of which had not previously been recognised in the glass making traditions of the Mediterranean. While the chemical characteristics of the late antique and early medieval fragments confirm the current model of glass production and distribution at the time, the elemental make-up of the majority of the eighth- to fourteenth-century glasses from Pergamon indicate the existence of a late Byzantine glass type that is characterised by high alumina levels. Judging from the trace element patterns and elevated boron and lithium concentrations, these glasses were produced with a mineral soda different to the Egyptian natron from the Wadi Natrun, suggesting a possible regional Byzantine primary glass production in Asia Minor.

## Introduction

The elemental composition of glass reflects the raw materials and the techniques that were employed in its manufacture. The chemical analysis of glass can therefore provide evidence about the origin of the raw materials, while the comparison of compositional data between archaeological sites can potentially reveal patterns in the production and the trade of glass. This in turn can shed light on the economic and cultural connections linking any one specific site to the wider world. Hence, the analytical study of archaeological glass can contribute substantially to our understanding of technological and cultural processes.

Yet, one of the most remarkable characteristics of Roman and early medieval glass (up to the 9^th^ century CE) from the Mediterranean is its compositional homogeneity. It is a soda-lime-silica glass with little variation in its major and minor element composition. The typically low levels of potassium and magnesium oxides (<1%) are usually attributed to the use of a pure form of an evaporate mineral soda (so-called *natron*), most likely from the Wadi Natrun in northern Egypt [1]. It is believed that these mineral soda glasses were produced on a very large scale in a limited number of primary glassmaking installations from two ingredients alone, namely imported *natron* (fluxing agent) and a silica source (network former) that was locally available. This implies that the remaining elements were introduced as contaminants of the main two ingredients. For example, the third most abundant component in ancient glass, calcium oxide (stabiliser), is present in the form of seashells or limestone in sands used as silica source. The so produced raw glass was then broken up into chunks and distributed to numerous secondary workshops throughout the Mediterranean as well as central and northern Europe, where the glass was remelted, colourants and/or opacifiers were added as required and where the glass was finally worked into artefacts [2]–[8]. There is ample evidence for the large-scale production of glass in the form of enormous glassmaking furnaces in Greco-Roman Egypt [9], [10] and in the late antique and early medieval Levant [11], [12]. Several shipwrecks from the same period contained substantial amounts of glass ingots and thus attest to the far-flung maritime trade in glass as well as to the division of labour [13]–[15]. However, evidence comes above all from the chemical makeup of glass assemblages from throughout the Mediterranean and Europe that can be related back to the known primary glass production groups in the Levant and Egypt (e.g. [3], [5], [16]). There may have been some primary glass production in western Europe during the Roman period [17]–[20]. Natural variations in the composition of the raw materials of glass over time can possibly account for some of the observed minor chemical differences [19], [21]. Similarly, technical factors inherent in the glass manufacturing processes have been shown to considerably impact the final glass composition [22], [23]. In any case, most of the analytical data to date indicate an eastern Mediterranean origin for the majority of first millennium CE glass.

The manufacture of *natron*-based glass appears to have ceased during the eighth/ninth century CE, when soda-rich plant ash at first complemented and eventually completely replaced the use of *natron* across the Islamic world, in the Near East (e.g. at al-Raqqa in Syria [24]) as well as in Egypt [25]. Simultaneously, medieval Europe saw the emergence of wood-ash glasses (e.g. [26]). The chemistry of plant or wood ash glasses is more complex than that of natron glasses as unrefined ashes can be highly variable, especially with regard to their sodium, potassium, magnesium and calcium oxide concentrations. Depending on whether crushed quartz pebbles or sand was used as the silica source, calcium oxide is introduced into the glass batch either exclusively as part of the plant ash (in case of quartz), or with both, ash and sand (shells or limestone). This can potentially result in the failure of the melt due to excessive lime levels [27]. The replacement of mineral soda with plant ash as fluxing agent for the manufacture of vitreous materials was presumably the direct consequence of shortages in the supply of Egyptian *natron*, possibly due to increasing demands, climatic factors or political disturbances in the Nile Delta [1], [28].

This concept of radical technological change in the manufacture of glass rests on analytical data limited both in chronological and geographical scope. Scholarly focus to date has been on the late antique and early medieval glass from the Middle East and Egypt [2], [9], [13]–[15], [24], [25], [29], [30]. Little is known, however, about the manufacture of Byzantine glass and its relationship to the glass making traditions of the eastern Mediterranean, as there is a distinct lack of analytical data of glass from the heartlands of the Byzantine Empire. In order to shed light on the chemical and technological aspects of Byzantine glass production, this study presents the chemical data of Byzantine glass finds from the residential area at the ancient city of Pergamon (Asia Minor) dating to the fourth to fourteenth century CE. The material from Pergamon is of high scientific and historical interest, because the city of Pergamon remained largely under Byzantine rule until the fourteenth century and the assemblage spans the transitional phase with respect to the technology of glass production. It may thus be expected that the elemental compositions of the glass finds from Pergamon reflect the different major glass-making traditions (*natron* versus plant ash). The main objective of this study thus was to relate the analytical data of the glass from Pergamon to the current model of glass production and distribution in the Mediterranean during the period of interest and to specifically trace the transition in the consumption of glass at Pergamon from late antiquity up to the fourteenth century. A model for the supply and consumption of Byzantine glass at Pergamon was developed by comparing the generated analytical data with those of recognised primary glass production groups as well as with glass from other contemporary consumer sites. The known glass production groups of the eastern Mediterranean, however, could not account for the analytical results of most of the middle and late Byzantine glass fragments from Pergamon, strongly indicating the existence of Byzantine primary glass production centres, possibly in Asia Minor itself.

## Materials and Methods

### Byzantine Pergamon and Its Glass

The fate of post-Roman Pergamon remains obscure and the archaeological and historical records are particularly scarce for the late antique and early medieval periods. The once prosperous city seems to have been in steady decline from the late third century CE onwards. Some building activities during the early fifth centuries bear witness to a short-lived economic prosperity, but the recurring outbreaks of plaque in the sixth century probably reduced both the city's size and population [31]. Pergamon virtually ceased to exist for about two centuries as a direct result of the Arab conquest in 716 CE [31], [32]. There is some evidence for a Byzantine re-settlement under Leo VI (886–912 CE), but a more substantial recovery seems to have taken place only towards the end of the eleventh and the beginning of the twelfth century [31], [33]. This is when the fortifications were refurbished and expanded and new clusters of houses were built over the ancient ruins. The archaeological record reflects a continuous growth of the population and an increase in commercially used building complexes in the second half of the thirteenth century [31]. The Ottomans finally conquered Pergamon during the early decades of the fourteenth century [31], [33].

During the archaeological excavations of the residential area on the southern slopes of the ancient acropolis at Pergamon, conducted by the German Archaeological Institute between 1973 and 1993, a considerable collection of glass finds were uncovered, dating from the Hellenistic to the Islamic era of the city. Several pieces of evidence point to the trade of glass jewellery during the second half of the thirteenth century CE [31], and the presence of glass chunks among the late Byzantine finds confirms that secondary processing of glass took place at Pergamon during this later period. No unambiguous evidence for the primary production of glass from its raw materials has been identified thus far. The glass fragments analysed in this study were excavated from a variety of contexts at the Byzantine levels and date to the fourth to fourteenth century CE. The set of samples consists of three pieces of glass chunks, five window fragments and twenty-three vessels including a rare prunted beaker (PEP-037), some painted or enamelled fragments (PEP-016, PEP-032, PEP-074) and two deep red opaque samples (PEP-043, PEP-096). Apart from the two red samples, all the vessels and windows are of a transparent or translucent quality and range from colourless, pinkish and aqua to darker shades of amber and green. Since the stratigraphic sequence is not ascertained beyond doubt [34], the artefacts were mainly dated on grounds of typological parallels [35, Schwarzer (in preparation) Antikes, byzantinisches und islamisches Glas aus Pergamon. Pergamenische Forschungen]. The assemblage was classified according to their typological attribution into three main archaeological phases: early Byzantine (4^th^–7^th^ century), middle Byzantine (8^th^/9^th^ century) and late Byzantine (12^th^–14^th^ century).

### Analytical Methods

For electron microprobe analysis (EPMA), small sections (about 1–2 mm^3^) were removed from the individual glass samples and mounted in epoxy resin blocks that were subsequently ground and polished down to 1 µm grade. The polished resin blocks were then coated with a thin conductive carbon layer. The chemical analysis of the major and minor components was carried out with a Jeol 8600 electron microprobe equipped with four wavelength-dispersive spectrometers (WDS) in the Research Laboratory for Archaeology and the History of Art at the University of Oxford. The operating conditions were a 15 keV accelerating voltage and a 6 nA incident beam current with the electron beam defocused at 10 µm and counting times of 30 s on peak and 15 s on background for the major and minor elements. 22 elements were analysed (Na, K, Mg, Ca, Ba, Ti, Cr, Mn, Fe, Co, Ni, Cu, Zn, Al, Si, Sn, Pb, P, As, Sb, S, Cl) and converted into weight percent (wt%) oxide values using the PAP correction programme (table 1). To ascertain the homogeneity of the samples and to obtain more representative results several measurements were taken of each sample and the mean calculated (n≥5). The precision (defined as the relative standard deviation σ/A, where σ is the standard deviation and A is the arithmetic mean) was within 1% for SiO_2_ and within 2% for Na_2_O and CaO, at about 3–4% for MgO, K_2_O and Al_2_O_3_ and typically within 10% for TiO_2_, MnO and Fe_2_O_3_. The detection limit was about 0.04 wt% for Cl and 0.07 wt% for P_2_O_5_ and TiO_2_. For all other trace elements, values below 0.1 wt% were below the detection limit and not taken into account. The accuracy of the measured data was evaluated against Corning ancient glass standards A and B (Table S1). To evaluate the major and minor elements in relation to the sand and alkali source (i.e. the base glass composition) and for comparison with published data the measured alumina and lime concentrations were corrected by +5% and by −10% relative, respectively. These corrections brought the measured results in line with the certified values of the Corning standards, making the data directly comparable to other published results. The glass compositions were then reduced to the seven main constituents SiO_2_, Na_2_O, CaO, MgO, K_2_O, Al_2_O_3_ and Fe_2_O_3_ and normalised to a sum of 100% as discussed by Brill [36].

**Table 1:** Results from the EPMA analysis (n≥5; values given in wt% of oxides) divided into chronological and chemical sub-groups.

Sample	Colour	Artefact	SiO_2_	Na_2_O	K_2_O	CaO	MgO	Al_2_O_3_	FeO	TiO_2_	MnO	P_2_O_5_	Cl	SO_3_	CuO	Sb_2_O_5_	PbO	Sum
***Early Byzantine natron-type glasses***																		
PEP_028	colourless	vessel	71.70	17.22	0.49	6.24	0.38	1.78	0.29	0.08		0.03	1.09	0.28		0.89		100.48
PEP_033	blue	vessel	69.61	15.14	0.50	8.20	0.47	2.35	0.77		0.42	0.16	0.96	0.24		2.05	0.58	101.49
PEP_063	colourless	vessel	71.18	18.03	0.44	6.09	0.50	2.00	0.34	0.08		0.02	1.26	0.25		0.58		100.76
PEP_064	light blue	window	67.21	14.99	0.70	8.55	0.63	2.55	0.68		0.65	0.18	0.89	0.20	0.37	0.98	1.78	100.35
PEP_065	aqua	window	73.49	14.54	0.53	7.26	0.44	3.13	0.49	0.12		0.05	0.79	0.15				100.99
PEP_066	aqua	vessel	66.27	14.51	0.57	8.43	0.62	2.43	0.99	0.09	0.53	0.16	0.67	0.21	0.52	1.29	2.93	100.21
PEP_085	aqua	vessel	66.52	18.00	0.78	8.60	0.95	2.50	0.85	0.16	0.79	0.15	0.84	0.21				100.37
PEP_099	greenish	window	65.85	18.57	0.49	6.27	0.89	2.53	1.20	0.47	2.64	0.05	1.00	0.28				100.23
PEP_100	colourless	window	68.68	16.23	0.73	9.10	0.57	2.81	0.35	0.07	1.15	0.09	0.94	0.21				100.93
***Middle Byzantine high alumina glasses***																		
PEP_043	dark opaque red	vessel	56.67	14.48	2.14	7.36	2.24	11.03	2.74	0.72		0.28	0.99	0.16	0.67			99.48
PEP_053	amber	vessel	55.34	17.82	1.78	5.32	1.58	9.90	1.95	0.63	3.66	0.36	0.95	0.15				99.44
PEP_096	dark opaque red	vessel	57.64	18.47	1.82	4.73	1.47	9.90	2.12	0.68	0.16	0.32	1.19	0.16	1.50			100.16
***Late Byzantine high alumina glasses***																		
PEP_015	dark green transl.	vessel	56.51	17.26	1.77	5.03	1.42	9.74	1.60	0.65	2.95	0.36	0.98	0.13				98.40
PEP_032	colourless	vessel	60.34	15.52	1.62	8.27	1.26	7.37	1.09	0.30	1.43	0.10	0.44	0.34				98.08
PEP_037	colourless	vessel	67.90	14.55	1.25	9.36	0.89	3.22	0.55	0.10	1.46	0.11	0.06	0.51				99.95
PEP_039	pinkish	vessel	65.11	14.05	2.06	9.14	0.84	5.88	0.64	0.12	1.28	0.10	0.08	0.51				99.80
PEP_047	yellowish pink	vessel	63.81	18.30	1.21	4.41	1.18	6.73	1.41	0.50	0.63	0.27	1.15	0.15				99.74
PEP_048	colourless	vessel	62.00	15.18	2.22	9.99	1.12	5.90	0.90	0.15	0.72	0.14	0.15	0.33				98.79
PEP_051	colourless	vessel	59.72	18.36	1.51	10.80	1.23	5.40	0.94	0.21	0.92	0.16	0.40	0.56				100.20
PEP_062	Red transl.	vessel	57.38	22.30	1.08	4.80	1.34	8.13	1.85	0.60	1.38	0.22	1.14	0.31				100.52
PEP_071	yellowish	vessel	57.85	19.25	1.27	5.34	1.57	9.77	2.16	0.82	0.59	0.24	1.14	0.06				100.05
PEP_078	colourless	vessel	64.00	15.82	1.58	9.64	1.02	5.31	0.66	0.11	0.56	0.06	0.08	0.46				99.31
PEP_081	pinkish	vessel	67.39	14.84	1.00	10.07	0.97	2.38	0.71	0.12	1.37	0.10	0.09	0.52				99.55
PEP_087	pinkish	window	61.50	16.60	1.75	10.09	0.99	5.20	0.51	0.12	1.56	0.08	0.07	0.53				99.02
PEP_088	dark olive	vessel	58.54	2.09	2.14	22.77	2.59	7.45	2.04	0.33		0.15	0.22	0.28				98.60
***Late Byzantine high magnesia (i.e. plant ash) glasses***																		
PEP_016	colourless	vessel	70.01	10.67	2.54	8.43	3.59	0.94	0.37	0.22	0.90	0.25	0.72	0.28				98.92
PEP_017	colourless	vessel	70.06	11.95	2.16	8.41	3.13	1.23	0.57	0.23	1.00	0.32	0.81	0.21				100.08
PEP_074	yellowish	vessel	69.03	12.00	1.69	10.33	2.55	1.45	0.97	0.25	1.60	0.35	0.90	0.16				101.26
***Late Byzantine glass chunks***																		
PEP_009	opaque olive	high Al	53.00	19.55	1.71	7.44	2.21	11.43	2.85	0.64	0.27	0.27	1.01	0.13				100.50
PEP_052	blue	high Mg	66.30	14.58	3.47	8.50	4.52	0.68	0.38			0.29	0.86	0.23				99.84
PEP_093	black	obsidian	75.14	4.16	5.02	0.80	0.12	13.49	0.92	0.14			0.05					99.83

The trace element composition of the glass fragments was determined by laser ablation-inductively coupled plasma-mass spectrometry (LA-ICP-MS) in the Field Museum at Chicago. The analytical parameters and protocol of the procedure have been previously described in detail [37]. In short, the trace element data are the result of an average of four measurements per mounted sample (table 2), taken with a laser beam diameter of 55 µm at 70% of the laser energy (0.2 mJ) at a frequency of 15 Hz. The laser ablation analyses were performed in helium as the carrier gas after a pre-ablation time of 20 s that served to eliminate the transient part of the signal and any possible surface corrosion or particles. ^29^Si was used for internal standardization and data were calibrated against two standard reference materials (NIST SRM 610, 612) and Corning B, C and D. The elemental compositions were then calculated according to Gratuze [38]. Detection limits range from 0.01–1 ppm for most of the elements, while the accuracy varies from 5% to 10%, depending on the elements and their concentrations [37]. Major and minor elements were also analysed by LA-ICP-MS and were in line with the results obtained by EPMA (variation for silica of 2%, sodium 5.5%, potassium and calcium oxides 6%, magnesia 8%, alumina 10%, manganese 12% and iron oxides 15%).

**Table 2:** Trace element results from the LA-ICP-MS analysis (n = 4; values given in ppm) divided into chronological and chemical sub-groups.

Sample	Li	Be	B	Sc	Ti	V	Cr	Ni	Co	Zn	As	Rb	Sr	Zr	Nb	Ag	Sb	Cs	Ba	La	Ce	Pr	Y	Bi	U	W	Nd	Th
***Early Byzantine natron-type glasses***																												
PEP_028	3	0.3	127	5	296	6	6	3	0.9	13	0	6	397	34	1.3	0.1	3994	0.1	126	5	8	1.1	5	0.0	1	0	4	0
PEP_033	3	0.9	117	4	206	15	12	24	458.2	32	10	7	441	40	2.1	1.3	10581	0.5	230	7	12	2.1	9	0.4	1	0	7	1
PEP_063	3	0.3	167	4	315	8	6	3	1.2	15	0	5	344	37	1.6	0.1	2567	0.0	124	5	9	1.3	5	0.0	1	0	5	1
PEP_064	4	0.3	137	3	307	19	9	21	101.4	100	8	8	429	35	1.8	2.5	6491	0.1	240	6	11	1.4	6	1.5	1	0	6	1
PEP_065	5	0.4	50	4	400	10	9	5	2.2	8	0	9	329	40	2.0	0.0	45	0.1	210	6	13	1.6	7	0.0	2	0	6	1
PEP_066	4	0.3	127	3	331	17	9	16	169.3	93	6	7	438	42	1.8	2.8	5922	0.1	239	7	11	1.6	7	1.3	1	0	6	1
PEP_085	7	0.8	149	5	679	27	15	14	12.0	31	0	9	546	65	3.0	0.8	50	0.5	260	7	13	2.3	8	0.4	2	1	7	2
PEP_099	5	0.4	188	6	1232	50	50	16	11.0	27	5	5	424	173	5.2	0.2	5	0.1	1236	8	15	2.0	9	0.1	1	1	8	2
PEP_100	3	0.6	108	3	199	17	10	6	5.2	13	3	10	446	33	1.5	0.0	1	0.1	432	6	12	1.5	7	0.0	1	0	6	1
***Middle Byzantine high alumina glasses***																												
PEP_043	26	2.4	580	11	1965	60	97	61	10.0	40	169	55	230	311	16.1	2.9	41	2.5	369	29	52	6.8	33	0.9	2	2	25	7
PEP_053	23	1.9	954	8	2621	57	85	43	6.3	672	524	36	408	266	14.3	1.1	196	2.4	733	27	54	6.8	27	0.0	5	2	25	9
PEP_096	19	1.8	694	7	1952	66	81	39	7.9	31	214	47	156	237	15.1	6.2	19	2.1	514	26	54	6.6	25	5.4	3	2	24	7
***Late Byzantine high alumina glasses***																												
PEP_015	*18*	*2.2*	*941*	*9*	*3123*	*310*	*83*	*40*	*26.7*	*41*	*227*	*35*	*207*	*279*	*16.4*	*0.2*	*3*	*1.4*	*5259*	*29*	*59*	*7.2*	*29*	*0.0*	*7*	*49*	*26*	*8*
PEP_032	258	3.3	1337	8	1641	153	44	22	21.2	34	6	71	1986	169	14.3	0.2	15	22.4	2766	19	41	4.8	22	0.2	8	7	17	11
PEP_037	303	2.6	1433	5	308	226	9	7	20.0	29	27	75	2895	31	3.9	0.2	42	44.1	2977	8	15	1.6	5	0.1	2	23	6	5
PEP_039	277	2.9	1367	5	366	123	9	6	14.4	31	34	112	2517	43	9.9	0.2	10	49.5	2714	13	57	3.0	11	0.1	4	14	10	9
PEP_047	16	1.5	1066	7	1264	67	70	26	9.1	22	1451	29	161	232	10.8	0.1	1	1.2	484	20	41	5.0	22	0.1	5	2	18	5
PEP_048	339	2.9	1451	5	363	66	10	9	13.0	26	28	117	2663	49	10.2	0.2	8	49.7	1139	12	27	2.6	9	0.1	4	19	9	10
PEP_051	300	3.0	1362	6	791	82	30	12	19.9	27	242	87	3079	129	10.0	0.1	8	44.9	1973	15	30	3.5	17	0.1	3	9	13	9
PEP_062	17	1.1	657	6	1909	43	66	27	4.8	44	341	20	193	215	10.3	0.2	578	0.8	249	20	42	5.1	20	0.0	14	1	19	6
PEP_071	24	2.2	712	9	3757	82	103	34	15.6	31	140	25	221	400	17.7	0.2	2	1.2	1334	36	74	9.1	35	0.1	6	1	33	12
PEP_078	352	2.7	1784	5	592	66	6	7	6.3	26	0	97	2644	35	8.4	0.1	9	54.1	1139	10	26	2.3	7	0.1	5	14	7	12
PEP_081	388	3.0	1810	5	587	169	8	7	15.9	24	2	66	3120	28	3.4	0.1	14	52.5	2754	8	15	1.7	5	0.0	1	24	6	3
PEP_087	438	3.9	1722	5	447	172	6	7	14.4	35	0	112	2979	27	8.7	0.1	9	50.0	2789	8	24	2.0	9	0.2	4	15	7	9
PEP_088	32	2.0	51	8	1705	42	61	23	5.9	53	0	64	382	182	7.9	0.2	1	2.3	286	25	48	6.3	21	0.2	3	1	23	9
***Late Byzantine high magnesia (i.e. plant ash) glasses***																												
PEP_016	6	0.9	70	5	1047	20	16	9	7.2	69	0	9	541	125	3.6	0.1	0	0.2	359	6	13	1.6	7	0.0	1	1	6	1
PEP_017	6	0.8	57	5	1072	18	19	9	7.8	94	0	9	554	119	3.5	0.2	0	0.2	208	6	12	1.5	7	0.1	1	0	6	1
PEP_074	8	1.2	55	5	809	23	23	14	6.7	131	2	8	505	118	4.2	0.1	1	0.4	234	7	14	1.7	10	0.0	2	0	7	1
***Late Byzantine glass chunks***																												
PEP_009	24	2.2	1424	11	3156	78	92	50	9.3	38	259	34	188	282	14.6	0.1	1	1.3	537	29	58	7.2	29	0.2	9	2	26	10
PEP_052	11	0.2	93	3	139	6	3	5	58.6	98	0	10	544	8	0.5	0.1	2	0.1	44	2	3	0.4	2	0.0	0	0	1	0
PEP_093	63	3.0	32	4	451	3	3	2	0.3	39	5	173	90	96	27.1	0.1	1	7.7	390	39	74	6.3	9	0.5	6	3	18	18

Multivariate statistical analysis of a subset of the data was carried out using SPSS 11 software to compare the analysed samples with published data. In order to identify and explain group structures, principal component analysis (PCA) was performed on the base glass data of the high alumina glasses (SiO_2_, Na_2_O, CaO, MgO, K_2_O, Al_2_O_3_ and Fe_2_O_3_). Additionally, PCA was done on trace elements that are likely to have been introduced to the glass with the silica or the alkali source (Rb, Sr, Y, Zr, Ba, La, Ce, Pr) rather than with any additives. Uranium was included in these analyses to discriminate between different high alumina glass groups as had been suggested previously [39], [40]. To minimise the errors inevitably caused by the difference in concentration the data were initially subjected to auto scaling. PCA was carried out on the matrix of correlation coefficients of each of the variables with every other variable. The relationships between the most expressive principal components as defined by an *Eigenvalue* of 1 or more was then determined on the basis of simple bivariate plots [41].

## Results

**Figure 1:**
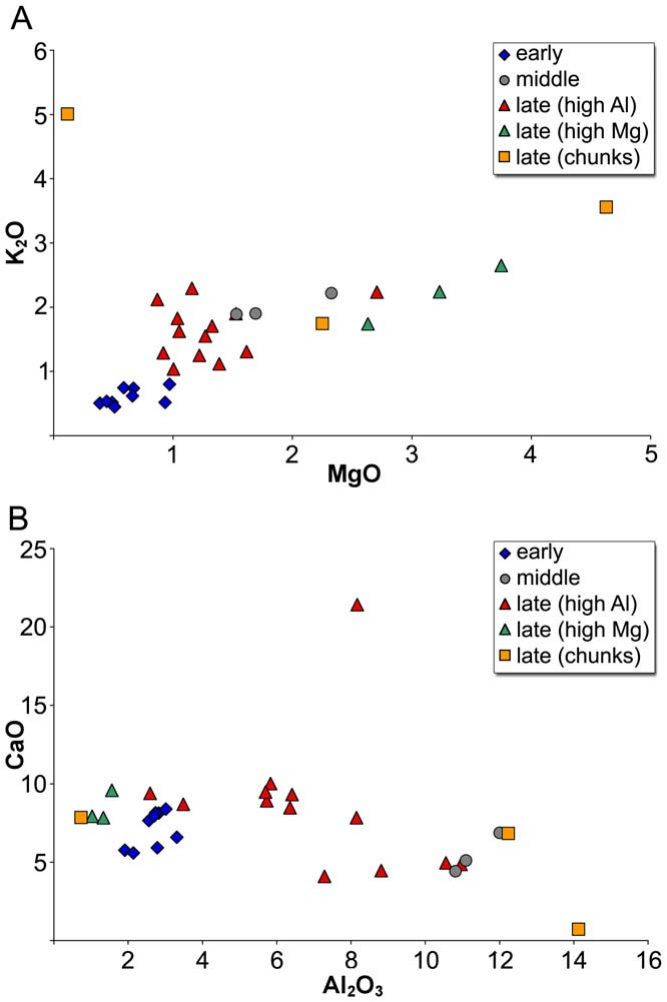
Major element compositions of 31 analysed glass fragments from Pergamon. (A) The comparison of potassium and magnesium oxide concentrations highlight the use of different sources of alkali for the production of different glass groups. The early samples from Pergamon (4^th^–7^th^ c., blue diamonds) show low levels of magnesia and potash (≤1%), indicating the use of mineral soda. Three late Byzantine samples (12^th^–14^th^ c., green triangles) have high and positively correlated potassium and magnesium oxide concentrations, implying the use of plant ash. Most of the middle (8^th^/9^th^ c., grey circles) and late samples (12^th^–14^th^ c., red triangles), however, have intermediate potash and magnesia levels (1–2%). Data from analysed glass chunks are shown as yellow squares. (B) Calcium and aluminium oxide contents confirm the division of the Pergamon assemblage into sub-groups. The early Byzantine glasses (blue diamonds) define a relatively narrow cluster, the late plant ash glasses (green triangles) are characterised by very low alumina concentrations, while the majority of the middle (grey circles) and late (red triangles) fragments have significantly increased alumina concentrations.

The major and minor element compositions of the analysed glass fragments from Pergamon indicate that with the possible exception of one vessel (PEP-088) the glass is of the soda-lime-silica type (table 1). Based on the magnesium, potassium and aluminium oxide contents the assemblage can be divided into at least three distinct groups (Fig. 1a&b). The first group comprises all the samples associated with the early Byzantine period and has magnesium and potassium oxide levels below 1%, suggesting that these samples were produced using a mineral source of soda. A second group has both oxides at concentrations between approximately 1% and 2% and cannot be unambiguously classified as mineral soda, mixed natron-plant ash or plant ash on grounds of the major and minor element composition alone. Most of the middle and late Byzantine fragments belong to this intermediate group. The samples in this group also show very high aluminium concentrations (Fig. 1b). Finally, a set of seven samples has magnesia contents in excess of 2% and potash ranging from 1.7% to 3.5%, which may be indicative for the use of plant ash. Yet, only three vessels (PEP-016, -017, -074) and possibly one glass chunk (PEP-052) can be singled out by their strong positive correlation between potassium and magnesium oxides (Fig. 1a) and above all on account of their substantially lower alumina levels than the other late Byzantine glasses (Fig. 1b). Two of these three thirteenth-century vessels (PEP-016 & PEP-074) exhibit painted decorations that can be attributed to the Mamluk period [35]and their chemical characteristics correspond to typical Islamic soda plant ash glass. This group of samples will therefore be examined separately in the discussion of high magnesia glasses. The remaining three samples with levels of magnesia in excess of 2% also display high alumina concentrations and are therefore discussed together with the other high alumina glasses (Fig. 1b).

Before elaborating on the individual glass groups in more detail, it is necessary to comment briefly on the vessel fragment PEP-088 and on the chunk glass PEP-093 as they will not feature again in this study. Its chemical characteristics identify sample PEP-093 clearly as a lump of black obsidian (e.g. [42]). The translucent dark olive green bottle PEP-088, attributed to the late Byzantine phase, is exceptional in its exceedingly high lime concentrations (∼23%) that are reminiscent of medieval European wood ash glass. However, the low levels of potash (∼2%) and the high alumina contents (∼7.5%) of PEP-088 are not compatible with the typical composition of European wood ash glasses (10-20% K_2_O and 1–3% Al_2_O_3_; see [26]). Instead, the sample's aluminium oxide and trace element pattern are closely related to the coloured high alumina glasses in the Pergamon assemblage. This sample may thus represent a sub-type of these glasses, whose lime content was drastically increased, possibly through wood ash contamination, the melt's reaction with the parting layer of the crucible or a combination of both factors (table 1&2) [22].

### Early Byzantine natron-type glass

The group of nine samples attributed to the early Byzantine period seem to define a relatively homogeneous group in terms of their lime, alumina, magnesia and potash concentrations. The generally low levels of potassium and magnesium oxides clearly point to the use of a mineral source of soda (Fig. 1a). Upon closer inspection, however, some variations are noticeable in the lime concentrations of these samples (Fig. 1b). Five samples form a very tight cluster with lime concentrations between 8% and 9% and alumina levels between 2.3% and 2.8%. The other four samples have notably lower lime levels (∼6–7%). Given that the lime and alumina concentrations are diagnostic of the silica source, these differences strongly imply that the early Byzantine glasses at Pergamon originated from more than one silica source, possibly indicating slight chronological differences.

**Figure 2:**
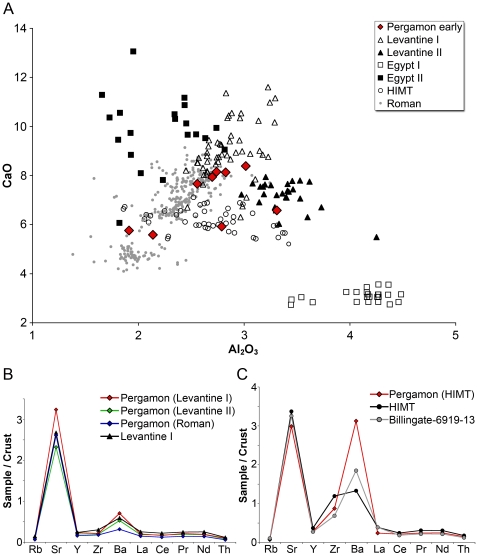
Early glasses from Pergamon in comparison with contemporary glass production groups. (A) The lime and alumina concentrations of the early Byzantine samples from Pergamon (red diamonds) were compared to the data of contemporary glass types (Roman glass from Italy [57]–[61]; Levantine I glass from Apollonia, Dor and Jalame; Levantine II glass from Bet Eli'ezer; HIMT glass from Billingsgate, Augusta and northern Sinai [by courtesy of Ian Freestone]; Egypt I and II glass [B. Gratuze, unpublished dissertation, University of Orleans, 1988: *Analyse non destructive d’objets en verre par des mŽthodes nuclŽaires*. *Application ¯ l’Žtude des estampilles et poids monŽtaires islamiques*]). Five Pergamon samples overlap with Levantine I glass (empty triangles), one sample with Levantine II (black triangles), one with HIMT (circles), and two fragments with first- to fourth-century Roman glasses (grey circles). (B) Concentrations of trace elements of the early Pergamon glasses, normalized to the mean continental crust [43]. The mean value of five Levantine I samples from Pergamon (indicated in red), the Levantine II sample (green) and the average of the two Roman glasses from Pergamon (blue) are compared to the average trace element pattern of 9 Levantine I glasses (black) from Apollonia [29]. (C) The trace element composition of the HIMT glass from Pergamon (shown in red) exhibits a similar pattern as the average trace element distribution of HIMT glasses (black) and in particular with an HIMT glass from Billingsgate (grey; [29]).

This interpretation can be further corroborated by superimposing the data from Pergamon on those of the different primary glass production groups that have been identified in the eastern Mediterranean during the period of interest (Fig. 2a). The cluster of five glasses seems to be associated with Levantine I glass, two samples correspond closely to somewhat earlier Roman glass from the first to fourth centuries, one window glass fragment is a clear example of HIMT glass while another window glass sample appears to be related to the Levantine II type. In order to substantiate these observations, we performed trace element analysis of the samples to clarify their affiliation with the different glass reference groups. Following the protocol introduced by Freestone and colleagues [5], [8] those trace elements were chosen that are believed to reflect the silica source used in the production of the base glass rather than other additives such as colourants or opacifiers. The data were then normalised against the average composition of the weathered upper continental crust [43]. The trace element compositions of the five samples that appear to be associated with Levantine I glass were averaged and represented as one mean trace element profile (Fig. 2b). This composition was compared to the mean of the two Roman fragments (PEP-028, -063), to sample PEP-065 that was attributed to the Levantine II type and to sample PEP-099 that was identified as HIMT glass (Fig. 2b&c). The compositional profiles of all but the HIMT sample are very similar and show consistently low levels of most trace elements. The sole exceptions are strontium and barium that are either close to or up to three times higher than the mean continental crust (Fig. 2b). This pattern strongly implies the use of mineralogically mature sand that is rich in quartz and low in heavy minerals and simultaneously high in strontium [8]. Such a trace element distribution is in fact characteristic of Levantine coastal sands as well as first millennium glasses from the Levant [3].

Comparing the trace element profiles of the early Byzantine samples from Pergamon with those of the Levantine I glasses from Apollonia suggests that apart from the HIMT specimen all natron-type glasses from Pergamon were made from the same or a very similar silica source as the one utilised for the Levantine glasses (Fig. 2b). There is a slight but noticeable difference between the Levantine and the Roman glasses. The Roman samples are on average more depleted in their trace elements, indicating an even purer silica source than the one used for the Levantine glasses. Nonetheless, their overall trace element distribution is closely similar and since some local variations can be expected, it is likely that all of these glasses were made with sand from the coastal stretch between the Nile and northern Israel [16]. Our data thereby confirm that the samples that otherwise exhibit the well-recognised characteristics of Roman glass were also made from Levantine coastal sands [9], [44]. The trace element pattern of sample PEP-099 differs from the other natron-type glasses in that it shows higher zirconium and barium concentrations (Fig. 2c). This is broadly in line with the pattern observed for HIMT glasses (e.g. an HIMT sample from Billingsgate), although the zirconium and barium contents of HIMT glasses are highly variable [16]. Nonetheless, the elevated iron, manganese and titanium levels of PEP-099 (table 1) unambiguously identify this sample as HIMT glass. In summary, the compositional characteristics of the early Byzantine samples from Pergamon resemble those of typical soda-lime-silica glasses from the south-eastern Mediterranean coast. While the majority of the fragments correspond relatively closely to the Levantine I type, there appears to be also a component of possibly earlier Roman as well as HIMT and Levantine II glass among the Pergamon assemblage. Significant levels of antimony and lead oxide in three of the five Levantine I fragments and their slightly elevated concentrations of cobalt, iron, manganese and titanium provide some evidence of recycling (table 1). These samples also show somewhat higher phosphorous values, which might indicate prolonged or repeated exposure to fuel ash and vapour during recycling [45]. Given the presence of antimony oxide, it is likely that recycled Roman glass constitutes a considerable proportion of the material used to produce these fragments. These glasses have at the same time on average higher iron, manganese and titanium contents relative to Roman or in fact Levantine I glass, suggesting the admixture of a glass type different to typical colourless Roman glass, either one similar to HIMT glass but without the increased zirconium levels, or coloured Roman glass with noticeable amounts of trace elements that ultimately derived from the colourants. The fact that two of these glass fragments are of a blue colour possibly indicates the use of Roman opaque blue mosaic tesserae, containing calcium antimonate as an opacifier and cobalt as the main chromophore (PEP-033, -064). Nonetheless, the close proximity of all the different natron-type glasses from Pergamon in terms of their trace element distribution strongly imply a common geographical region of origin for their silica source. This, however, does not mean that an identical silica source was used. The variations in the lime and alumina concentrations clearly show that different sands along the Levantine coast must have been exploited.

### High magnesia plant ash glasses

**Figure 3:**
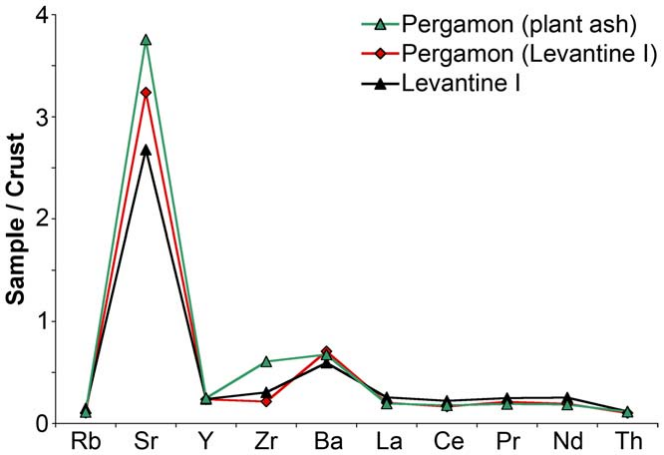
Trace element pattern of the plant ash glasses from Pergamon. Comparison of the average trace element distribution of the three plant ash glasses (shown in green) and the five Levantine I type fragments from Pergamon (red) with the mean of 9 Levantine I samples (black) from Apollonia [29]. The measured concentrations were normalised to the mean upper continental crust [43].

Three vessels were singled out on the basis of their high levels of magnesia (>2.5%) combined with elevated levels of potash (>1.5%) and phosphorous (0.25–0.35%). The compositional data indicate unequivocally that these high magnesium glasses were made using plant ash as the fluxing agent rather than the mineral soda employed in the early Byzantine glasses (Fig. 1a; table 1). The high magnesia glasses have also substantially lower alumina contents (∼1–1.5%) than the early Byzantine fragments (∼2–3%). Since the alumina level reflects the sand source, this difference would seem to suggest that the high magnesia glasses derived from a different silica source than the early Byzantine samples (Fig. 1b). It is widely assumed that Islamic plant ash glass was mostly produced with either relatively pure quartz-rich sands or crushed quartz pebbles [30]. Somewhat unexpectedly, however, the trace element pattern of these three high magnesia fragments resembles those of the early Byzantine glasses closely (Fig. 3). It deviates only in terms of somewhat increased levels of strontium and zirconium. While a significant proportion of the strontium was certainly introduced as part of the plant ash, the zirconium is likely to be derived from the silica source. In conjunction with considerable amounts of titanium oxide (0.2–0.25%) and manganese oxide (∼1%) a silica source similar to the one utilised for HIMT glass seems possible. This silica deposit must have been relatively poor in alumina and lime, as most of the calcium oxide in these glasses would have been introduced together with the plant ash.

That Islamic plant ash glasses of a similar low alumina concentration and overall chemical pattern as the ones described here were indeed produced from Levantine coastal sand has recently been convincingly demonstrated on the basis of strontium and neodymium isotope analyses [46]. The eleventh- to thirteenth century glasses from Banias have major and minor element compositions that are comparable to the three high magnesia glasses from Pergamon [29]. Degryse and colleagues have shown that the neodymium isotopic signature of these Banias glasses is consistent with Mediterranean coastal sands and that the silica for the Banias glasses therefore most certainly originated from the coastal stretch between the Levant and the Nile [46]. Judging from the close resemblance of the Pergamon and Banias plant ash glasses in terms of their major, minor and trace element characteristics, we therefore hypothesise that the Pergamon high magnesia glasses were made from a coastal sand, too.

### High alumina glasses

Disregarding two outliers (PEP-037, -081), the remaining eighth- to fourteenth-century samples contain intriguingly high amounts of aluminium oxide, ranging from about 5% to as much as 12% (Fig.1b). What is more, the magnesia and potash levels of these samples do not unambiguously determine whether a mineral or an organic fluxing agent was employed in their production (Fig. 1a). Compared to the so-called mixed alkali glasses (i.e. mixture of natron and plant ash) that have been identified among the eleventh-century glass from the Serçe Limani shipwreck, and the roughly contemporary mosaics at Torcello, Daphni and Hosios Loukas, the high alumina glasses from Pergamon show invariably higher sodium levels, while the magnesium, potassium and phosphorous oxide contents are markedly lower [36], [47], [48]. A similar mixture of mineral natron and plant ash for the Pergamon samples seems thus rather unlikely. The majority of the glasses have magnesia and potash levels below 1.5%, which is the generally accepted cut-off between mineral soda and plant ash glasses [49]. Even though this delineation may not always be accurate, the low phosphorous content of these glasses is consistent with that of the earlier mineral soda artefacts, supporting the hypothesis that a mineral alkali source was exploited for their manufacture. Based on their colour (colourless/pinkish versus coloured) and above all on grounds of their elemental composition (see below), the set of sixteen middle and late Byzantine samples can furthermore be separated into two sub-groups.

### Colourless and pinkish high alumina glasses

**Figure 4:**
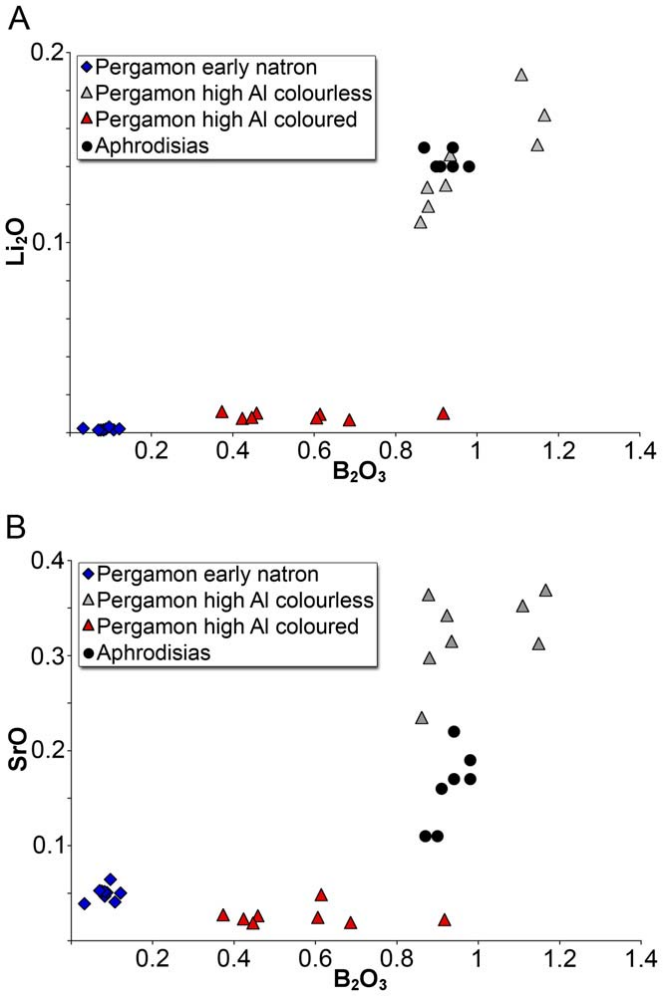
Trace element concentrations of the high alumina glasses from Pergamon. (A) The comparison of lithium and boron concentrations differentiates the colourless and pinkish high alumina glasses (light grey triangles), from the coloured high alumina glasses (red triangles) and the natron-type glasses from Pergamon (blue diamonds). The boron and lithium contents are positively correlated in the colourless and pinkish high alumina Pergamon samples, and are similar to the concentration in samples from Aphrodisias (black circles; [36], [54]. (B) The strontium levels further separate the colourless and pinkish (light grey triangles) and the coloured high alumina glasses (red triangles) from Pergamon. The fragments from Aphrodisias (black circles) show intermediate strontium levels. The early natron-type glasses from Pergamon (blue diamonds) are plotted for comparison.

**Figure 5:**
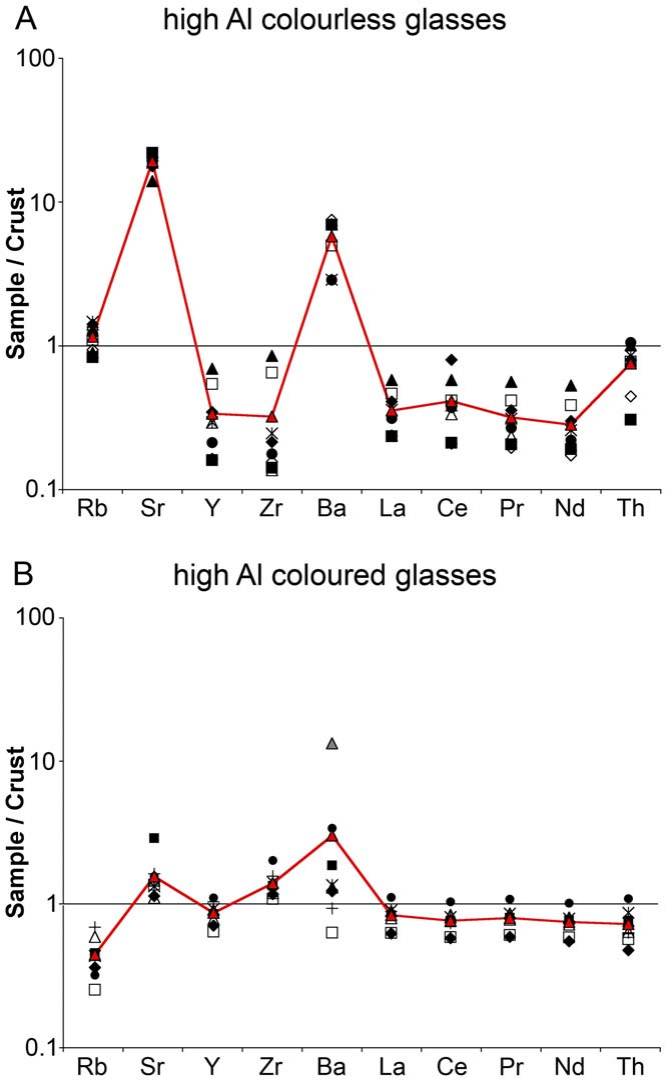
Trace element distributions of the high alumina glasses from Pergamon. (A) The trace element pattern of individual colourless and pinkish samples from Pergamon (black and open symbols) and the average concentration (red line) were normalised to the mean continental crust [43], and are displayed on a logarithmic scale. (B) Trace element distributions of the coloured high alumina glasses from Pergamon (black, grey and open symbols) and mean trace element pattern (red).

The eight colourless and faintly pinkish samples form a relatively homogeneous group, having an average of about 64% silica, 14–16% sodium, 9–10% lime and 5–6% alumina; potash levels vary between 1% and 2%, while magnesia is constant at about 1% (table 1). Interestingly, with the exception of sample PEP-032 the chlorine levels of these glasses are exceptionally low (<0.1%), which is highly unusual for ancient glasses. Also notable is the presence of titanium (0.1–0.3%) and manganese oxides (0.5–1.5%). The latter may indicate the deliberate addition of manganese as a decolourant to counteract the colouring effect of iron oxide (0.5%–1%). The most remarkable feature of all these glasses, however, is their extraordinarily high boron content (∼1% B_2_O_3_), which is about tenfold higher than the early Byzantine samples from Pergamon. The boron levels are positively correlated with high lithium concentrations (∼0.1% Li_2_O) that are about two orders of magnitude above the amount typically encountered in ancient glass (Fig. 4a). Boron and lithium are furthermore associated with considerable strontium levels (∼0.2–0.4% SrO; Fig. 4b) that lie about ten to twenty times above the average continental crust composition (Fig. 5a). Unusually high are also the caesium, barium and tungsten contents, while vanadium is only slightly elevated relative to the mean continental crust (table 2). Other than that the glasses are depleted in rare earth and trace elements, indicating the use of a mature silica source (Fig. 5a). The faint pinkish hue of three of the fragments (PEP-039, -081, -087) is probably the result of significant amounts of manganese oxide (1.3–1.6%).

### Coloured high alumina glasses

Seven vessels and one glass chunk belong to the group of coloured high alumina glasses. The colours represented are opaque and translucent deep red, translucent greenish yellow, amber and dark olive green. The composition of this set of samples is much more variable than that of the colourless high alumina glasses. The average silica concentration is about 58%, the soda contents range from about 14.5% to as much as 22%, lime varies between 4.5% and 7.5%, the alumina contents lie between about 7% and 11.5% and magnesia and potash lie between 1% and 2.2% (table 1). Most of these glasses show considerable concentrations of chlorine (∼1%), titanium (∼0.3–0.8%), manganese (∼0.2–3%) and iron oxide (∼1.5–3%). Given the colour palette of the samples, it is doubtful that manganese had any colouring or de-colouring effect in these glasses. The increased levels of iron oxide probably underlie the green and amber colours, while copper presumably in the form of cuprites is present in the opaque deep red fragments (PEP-043, -096). The boron content of this group of samples is also relatively high (0.05–1% B_2_O_3_), but the lithium levels do not stand out (≤0.01% Li_2_O) and at about 0.02% the strontium oxide concentration is much lower than in the colourless samples (Fig. 4a&b). Disregarding the transition metals that exhibit highly variable and increased profiles that can be attributed in most cases to the colourants, the trace element characteristics of these glasses are by far not as conspicuous as the ones of the colourless high alumina glasses (Fig. 5a&b, Table 2). On average, the zirconium levels are slightly increased compared to the colourless glasses, whereas the barium concentrations range from below the levels of the mean continental crust (<396 ppm) to as much as 0.6% in the case of sample PEP-015. This sample has extraordinarily high levels of tungsten (∼50 ppm). All other rare earth elements and trace elements associated with the silica source are generally depleted in this group of glasses when normalised against the average continental crust composition (Fig. 5b).

## Discussion

The analytical data of the 31 glass fragments excavated from the late antique to late Byzantine contexts at Pergamon have revealed the presence of three main primary glass compositions, within which several sub-groups can be identified. There were no surprises with respect to the early Byzantine mineral soda-type glasses that seem to represent three or four of the major primary production groups recognised in the south-eastern Mediterranean during the late antique and early medieval period. Equally, a plant ash recipe for some of the late Byzantine glasses at Pergamon was to be expected. Interestingly, these plant ash glasses were probably produced from coastal sand not so different from the earlier Levantine or HIMT natron-type glasses. What is most intriguing, however, is that only a very limited number of the eighth- to fourteenth century glasses (i.e. three specimens out of twenty one) are of a typical soda plant ash composition, while the vast majority of the middle and late Byzantine samples contain excessive amounts of alumina and could not be classified as plant ash glasses, given their overall low magnesium, potassium and phosphorous oxide levels. Instead, it seems rather likely that a mineral soda-rich efflorescence was used to produce these high alumina glasses. The use of mineral soda and concentrations of alumina in excess of 4% are very unusual for Mediterranean glasses of this period. Such a combination of mineral soda and high alumina is commonly associated with the Indian subcontinent and East Africa [37], [39], [50]. Only in recent years were high alumina glasses identified among medieval assemblages in central Jordan [51] or, in fact, among the eleventh- to thirteenth century glasses from Sardis about 100 km south-east of Pergamon as the crow flies [36], [39], [50].

No primary manufacturing centre for high alumina glasses is known to date. It has nonetheless been proposed that high alumina glasses originated ultimately in India where large amounts of mainly glass beads with high alumina concentrations are present in the archaeological record and where mineral soda (e.g. *reh*) and silica sources rich in alumina are readily available [36], [40]. The African high alumina glasses can in general be related to Indian or south-east Asian assemblages, thus supporting the theory of a south Asian source for these glasses. In contrast, the high alumina glass found at Sardis seems exceptional in that it was the only mineral soda and high alumina glass published by Dussubieux and colleagues that occurred exclusively outside south and south-east Asia [39]. Similarly, none of the Asian or African samples seem closely related to the high alumina bangles from Tell Abu Sarbut and Khirbat Faris [51]. However, these Jordanian assemblages are rich in potassium oxide (∼6–10%), indicating that they were made from plant ash. Given their very specific chemical fingerprint, these Jordanian glasses do not provide appropriate comparative data for the type of high alumina glasses retrieved from Pergamon.

**Figure 6:**
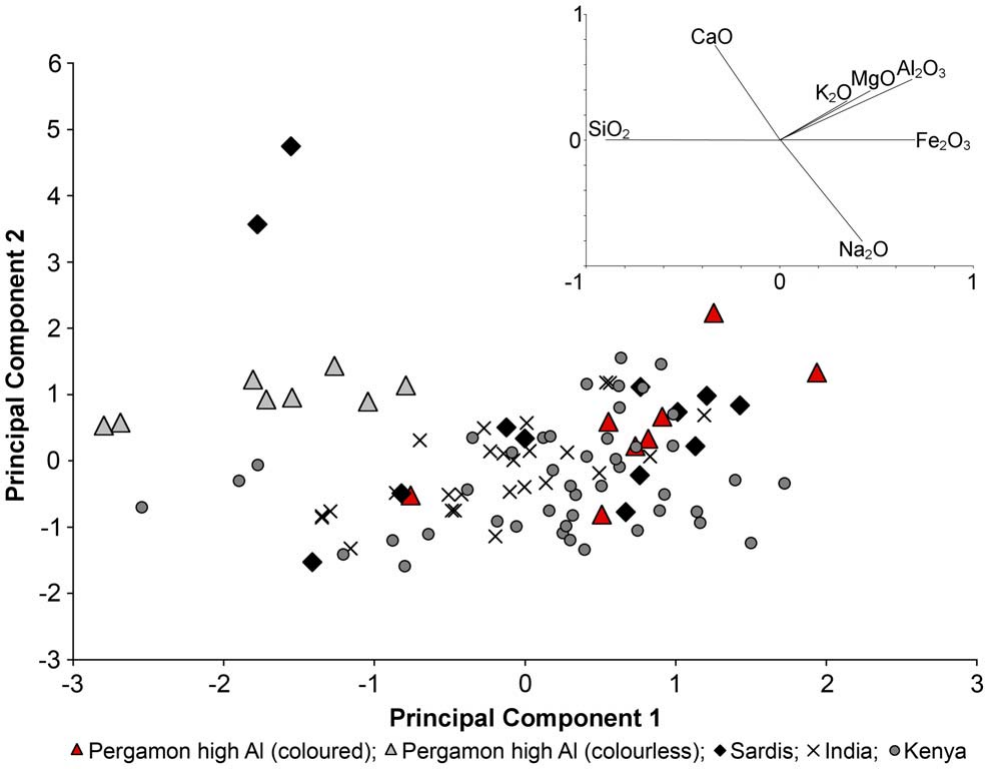
Principal component analysis of the major and minor elements. The high alumina glasses from Pergamon (colourless and pinkish samples represented as grey triangles; coloured samples shown as red triangles) were compared with data sets from mineral soda high alumina glasses from Sardis (black diamonds [36] and by courtesy of Laure Dussubieux), India (crosses) and Kenya (circles)[40], using principal component analysis (PCA) of the major and minor base glass elements (SiO_2_, Na_2_O, CaO, Al_2_O_3_, MgO, K_2_O, FeO). The loading vectors are shown to illustrate the multi-dimensional group structures (inset).

**Figure 7:**
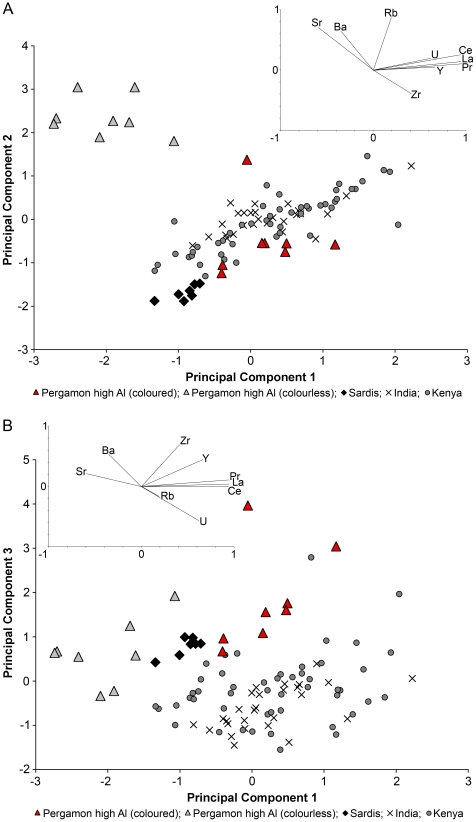
Principal component analysis of selected trace elements. The high alumina glasses from Pergamon, Sardis (by courtesy of Laure Dussubieux), India and Kenya [40] were analysed by PCA for the trace elements associated with the base glass composition (Sr, Ba, Rb, Ce, La, Pr, Y, Zr) in addition to uranium. Principal components PC1 and PC2 (A) or PC3 (B) distinguish the colourless and pinkish high alumina glasses from Pergamon (light grey triangles) from the other groups. The loading vectors illustrate that these samples have higher strontium and barium concentrations than the other groups. The coloured Pergamon samples (red triangles) overlap to a certain extent with the glass beads from Kenya (grey circles) and India (crosses). The samples from Sardis (black diamonds) contain particularly low levels of trace elements.

To clarify the relationship between the different high alumina glass assemblages we conducted a principal component analysis (PCA) of the seven base glass elements (SiO_2_, Na_2_O, CaO, Al_2_O_3_, MgO, K_2_O, FeO), comparing the high alumina glasses from Pergamon with glasses from India, Africa and Sardis. This analysis unequivocally singles out the colourless fragments from Pergamon, while the coloured samples overlap with the other groups to a certain extent (Fig. 6). The colourless glasses from Pergamon are distinctive in that they have higher silica and lime concentrations, while they are on average significantly lower in all the other base glass elements. The coloured specimens from Pergamon on the other hand seem to bear some resemblance to the east African assemblages and especially to the high alumina glasses from Sardis [36], [39], [40]. This overlap, however, can largely be resolved by exploring the trace element distribution of the individual groups by PCA (Fig. 7a&b). For this analysis those trace elements were chosen that reflect the base glass materials rather than any colourants and/or opacifiers (Rb, Sr, Zr, Ba, La, Ce, Pr and Y) in addition to uranium that has proved to be diagnostic for mineral soda high alumina glasses [39], [40], [50]. The analysis revealed that the colourless glasses from Pergamon are enriched in rubidium, strontium and barium (positively associated with PC2) and lower in all other trace elements compared to the other high alumina groups (Fig. 7a). The coloured glasses from Pergamon have consistently lower trace elements than the Indian and the majority of the Kenyan high alumina assemblages, with the exception of zirconium that is slightly increased in the coloured Pergamon samples (Fig. 7a&b). There might nonetheless be a certain overlap between the coloured high alumina glasses from Pergamon and glasses from the east coast of Africa, bearing in mind that the comparative Kenyan data that were available for the present study derive exclusively from glass beads and may thus not be representative of other glass artefacts. A typological link had previously been observed between glass vessels found in Sub-Saharan Africa and the Middle East, which is not entirely unexpected in light of Islamic trade routes that encompassed Kenyan coastal ports [39, Dussubieux & Kusimba (forthcoming) and references therein].

Despite these possible relationships, the most remarkable finding of our study is that the high alumina glasses from Pergamon represent two very distinctive compositional types neither of which has been identified before. The two groups are different from each other and, in fact, from the roughly contemporary assemblage from Sardis. The colourless/pinkish group differs as much in their major element composition from the other glasses (higher SiO_2_ and CaO; lower Na_2_O, Al_2_O_3_, K_2_O, MgO, FeO) as it shows a unique trace element pattern. The coloured glasses from Pergamon on the other hand resemble those from Sardis in terms of the major components, but can be distinguished from these with the help of their trace element distributions. The most unusual feature of both high alumina groups from Pergamon is their elevated boron concentration that is conspicuously absent from all other high alumina glass assemblages. Boron is particularly high in the colourless and pinkish fragments where it correlates positively with high lithium and strontium concentrations (Figs. 4a&b).

The considerable concentrations of boron may in fact provide clues about the raw materials used for the production of the Pergamanian high alumina glasses and, by extension, indicate the possible location of primary glass making centres during the Byzantine period. Although the possibility that boron was present as a contaminant in other raw materials such as the silica source cannot be excluded, boron is more generally associated with alkali and alkaline earth metals and has thus more likely been introduced with the alkali source. The use of plant ash as the fluxing agent in the production of these glasses was excluded on grounds of the relatively low levels of both magnesium and potassium oxides. The use of a mineral source of soda, however, is unprecedented for this time and place and needs further elaboration. It is widely assumed that a fairly pure mineral source of soda from the evaporitic mineral deposits in the Wadi Natrun in northern Egypt supplied most, if not all of the alkali for the Roman and early medieval glass production in the Mediterranean, the Near East and even Europe [1]. It is furthermore believed that this type of Egyptian *natron* was no longer employed from the late first millennium CE onwards and replaced by soda rich plant ash in the eastern Mediterranean and by potassium rich wood ash in Europe [1], [28]. Part of the reason for this radical change in the raw materials and glass production technology may have been that Egypt was no longer able to meet the increasing demands in mineral soda, possibly due to political disturbances [1]. Therefore, the manufacture of mineral soda glasses during the end of the first and the beginning of the second millennium CE, such as the ones in the Pergamon assemblage, clearly contradicts the current view on the history of glass production in the Mediterranean. What is more, the contamination levels of the glasses from Pergamon with various alkali and alkaline earth metals suggest the use of a fluxing agent different to the pure form of soda usually extracted from the Wadi Natrun. It thus seems necessary to consider possible alternatives of evaporitic sodium-rich deposits. A potential source might be the borate reserves in western Anatolia, not far from Pergamon, where the world's largest colemanite and ulexite deposits (Ca and Na-Ca borate formations) can be found [52]. These borate deposits are often associated with increased lithium and strontium levels, even though the exact ratios and absolute concentrations of these elements are highly variable [53]. Brill had linked fifth to seventh century CE glass from Aphrodisias in western Asia Minor to this local source of raw materials and speculated that either the salts from the colemanite deposits in the region or the ashes from plants growing there would probably result in high contaminations of boron, lithium and strontium [54]. The cullet and vessels from Aphrodisias indeed have concentrations of boron and lithium that are virtually identical with the colourless glasses from Pergamon and their strontium levels are likewise increased (Fig. 5a&b). It seems doubtful, though, that the use of plant ash would result in boron concentrations of nearly 1%, especially considering the toxicity of boron for plants and the ability of boron-tolerant plants to effectively efflux boron [55], [56]. As regards the high alumina glasses from Pergamon, we therefore propose the use of a soda-rich mineral containing boron in association with lithium and strontium as the fluxing agent, together with a silica source rich in alumina. Given that the elemental composition of evaporites can vary widely within the same deposit depending on the season, it is conceivable that the same or similar borate deposits were exploited for both the colourless and the coloured glasses from Pergamon. The silica source, however, cannot have been the same, as demonstrated by the alumina and trace element contents of the two sub-groups.

The chemical peculiarities of the high alumina glasses from Pergamon have far reaching consequences for our understanding of primary glass production towards the end of the first and the beginning of the second millennium CE. Firstly, the specific increase of a number of trace elements point to the exploitation of an evaporite rich in soda and contaminants and different to the one in the Wadi Natrun. Secondly, the two compositional sub-groups among the high alumina glasses strongly indicate the use of at least two different silica sources. If we assume that primary glass production centres were commonly located close to the silica source, this further implies the existence of more than one glass making factory that used this mineral source of soda and that supplied glass to Pergamon (and possibly Aphrodisias and Sardis). These findings provide incontrovertible evidence for the existence of an as yet unknown primary glass production group, that, judging from some parallels with analytical data from Sardis and Aphrodisias may have been typical of Asia Minor. This in turn points to a regional glass manufacturing tradition hitherto unrecognised.

It is conceivable that the Pergamon assemblage is the result of experimentation with new raw materials in response to the shortage of Egyptian mineral soda. That these events are indeed related is suggested also by the presence of the typical earlier Levantine and Egyptian primary glass production groups among the Pergamon assemblage. Only with the onset of the eighth century, the period in which we see changes in the use of raw materials in Europe and the Islamic Middle East and Egypt, do the high alumina glasses appear in the archaeological record of Pergamon. As such, the Pergamon samples may represent the Byzantine equivalent to the development of European and Islamic plant ash recipes. However, analytical data of glass from medieval Byzantium are relatively scant and more analytical work needs to be conducted on Byzantine glass assemblages from Asia Minor and potential silica and alkali sources in the region of the borate deposits. This might lead to the identification of the primary production centres of these glasses and further strengthen the concept of a Byzantine glass industry in Asia Minor that produced characteristic high alumina high boron glasses.

## Supporting Information

Table S1 **EPMA results for Corning glass standards (in wt% of oxides) to monitor the accuracy of the measurements.** Absolute deviations (measured – expected value, in wt%) and relative deviations (absolute deviation divided by the expected value, in %) are indicated.
(DOC)

## References

[1] Shortland A, Schachner L, Freestone I, Tite M (2006). Natron as a flux in the early vitreous materials industry: sources, beginnings and reasons for decline.. Journal of Archaeological Science.

[2] Freestone IC, Vandiver PB, Mass JL, Murray A (2005). The provenance of ancient glass through compositional analysis.. Materials Issues in Art and Archaeology VII.

[3] Freestone IC, Maggetti M, Messiga B (2006). Glass production in Late Antiquity and the Early Islamic period: a geochemical perspective.. Geomaterials in Cultural Heritage.

[4] Freestone IC, Greenwood R, Gorin-Rosen Y, Kordas G (2002). Byzantine and Early Islamic Glassmaking in the Eastern Mediterranean: Production and Distribution of Primary Glass.. Hyalos-Vitrum-Glass: Proceedings of the First Hellenic Glass Conference.

[5] Freestone IC, Hughes MJ, Cramp R (2006). The origins of the Jarrow glass.. Wearmouth and Jarrow Monastic Sites.

[6] Freestone IC, Jackson-Tal RE, Tal O (2008). Raw Glass and the Production of Glass Vessels at Late Byzantine Apollonia-Arsuf, Israel.. Journal of Glass Studies.

[7] Freestone IC, Leslie KA, Thirlwall M, Gorin-Rosen Y (2003). Strontium isotopes in the investigation of early glass production: Byzantine and early Islamic glass from the Near East.. Archaeometry.

[8] Freestone IC, Ponting M, Hughes MJ (2002). The origins of Byzantine glass from Maroni Petrera, Cyprus.. Archaeometry.

[9] Nenna M-D, Picon M, Vichy M, Nenna M-D (2000). L'atelier primaires et secondaires en Egypte a l'époque Greco-Romaine.. La route du verre.

[10] Nenna M-D, Vichy M, Picon M (1997). L'atelier de verrier de Lyon, du 1er siècle après J.-C., et l'origine des verres “Romains”.. Revue d'Archéométrie.

[11] Gorin-Rosen Y (1995). Hadera, Bet Eli'ezer.. Excavations and Surveys in Israel.

[12] Gorin-Rosen Y, Nenna M-D (2000). The ancient glass industry in Israel – summary of the finds and new discoveries.. La route du verre.

[13] Foy D, Picon M, Vichy M (2003). Verres Omeyyades et Abbasides d'origine Egyptienne: Les témoignages de l'archéologie et de l'archéométrie..

[14] Foy D, Picon M, Vichy M, Thirion-Merle V, Foy D, Nenna M-D (2003). Caractérisation des verres de la fin de l'Antiquité en Méditerranée occidentale: l'émergence de nouveaux courants commerciaux..

[15] Foy D, Vichy M, Picon M (2000). Lingots de verre en Méditerranée occidentale..

[16] Freestone IC, Hughes MJ, Stapleton CP, Evison VI (2008). The Composition and Production of Anglo-Saxon Glass.. Catalogue of Anglo-Saxon Glass in the British Museum.

[17] Degryse P, Schneider J (2008). Pliny the Elder and Sr-Nd isotopes: tracing the provenance of raw materials for Roman glass production.. Journal of Archaeological Science.

[18] Jackson CM, Joyner L, Booth CA, Day PM, Wager ECW (2003). Roman glass-making at Coppergate, York? Analytical evidence for the nature of production.. Archaeometry.

[19] Leslie KA, Freestone IC, Lowry D, Thirlwall M (2006). The provenance and technology of Near Eastern glass: Oxygen isotopes by laser fluorination as a complement to strontium.. Archaeometry.

[20] Wedepohl KH, Baumann A (2000). The use of marine molluskan shells for Roman glass and local raw glass production in the Eifel area (Western Germany).. Naturwissenschaften.

[21] Krom MD, Stanley JD, Cliff RA, Woodward JC (2002). Nile River sediment fluctuations over the past 7000 yr and their key role in sapropel development.. Geology.

[22] Rehren Th (2008). A review of factors affecting the composition of early Egyptian glasses and faience: alkali and alkali earth oxides.. Journal of Archaeological Science.

[23] Tanimoto S, Rehren Th (2008). Interactions between silicate and salt melts in LBA glassmaking.. Journal of Archaeological Science.

[24] Henderson J, McLoughlin SD, McPhail DS (2004). Radical changes in Islamic glass technology: Evidence for conservatism and experimentation with new glass recipes from early and middle Islamic Raqqa, Syria.. Archaeometry.

[25] Gratuze B, Barrandon JN (1990). Islamic Glass Weights and Stamps - Analysis Using Nuclear Techniques.. Archaeometry.

[26] Wedepohl KH, Simon K (2010). The chemical composition of medieval wood ash glass from Central Europe.. Chemie der Erde-Geochemistry.

[27] Freestone IC, Gorin-Rosen Y (1999). The great glass slab at Bet-She'Arim, Israel: An early Islamic glassmaking experiment?. Journal of Glass Studies.

[28] Whitehouse D (2002). The transition from natron to plant ash in the Levant.. Journal of Glass Studies.

[29] Freestone I, Gorin-Rosen Y, Hughes MJ, Nenna M-D (2000). Primary glass from Israel and the production of glass in late antiquity and the Early Islamic period.. La route du verre.

[30] Henderson J, Evans J, Barkoudah Y (2009). The roots of provenance: glass, plants and isotopes in the Islamic Middle East.. Antiquity.

[31] Rheidt K (1991). Altertümer von Pergamon, XV 2: Die Stadtgrabung. Die byzantinische Wohnstadt..

[32] Foss C (1977). Archaeology and the “Twenty Cities” of Byzantine Asia.. American Journal of Archaeology.

[33] Radt W (1999). Pergamon: Geschichte und Bauten einer antiken Metropole..

[34] Japp S (2010). Die sogenannte gilded ware - eine mutmasslich frühbyzantinische Keramikgefässgruppe in Pergamon.. Istanbuler Mitteilungen.

[35] Schwarzer H, Lafli E (2009). Spätantike, byzantinische und islamische Glasfunde aus Pergamon.. Late Antique/Early Byzantine Glass in the Eastern Mediterranean.

[36] Brill RH (1999). Chemical Analyses of Early Glasses..

[37] Dussubieux L, Robertshaw P, Glascock MD (2009). LA-ICP-MS analysis of African glass beads: Laboratory inter-comparison with an emphasis on the impact of corrosion on data interpretation.. International Journal of Mass Spectrometry.

[38] Gratuze B (1999). Obsidian characterization by laser ablation ICP-MS and its application to prehistoric trade in the Mediterranean and the Near East: Sources and distribution of obsidian within the Aegean and Anatolia.. Journal of Archaeological Science.

[39] Dussubieux L, Gratuze B, Blet-Lemarquand M (2010). Mineral soda alumina glass: occurrence and meaning.. Journal of Archaeological Science.

[40] Dussubieux L, Kusimba CM, Gogte V, Kusimba SB, Gratuze B (2008). The trading of ancient glass beads: new analytical data from South Asian and East African Soda-Alumina glass beads.. Archaeometry.

[41] Shennan S (1997). Quantifying Archaeology..

[42] Carter T, Poupeau G, Bressy C, Pearce NJG (2006). A new programme of obsidian characterization at Catalhöyük,Turkey.. Journal of Archaeological Science.

[43] Kamber BS, Greig A, Collerson KD (2005). A new estimate for the composition of weathered young upper continental crust from alluvial sediments, Queensland, Australia.. Geochimica et Cosmochimica Acta.

[44] Freestone IC, Martinón-Torres M, Rehren Th (2008). Pliny on Roman Glassmaking.. Archaeology, history and science: integrating approaches to ancient materials.

[45] Paynter S (2008). Experiments in the Reconstruction of Roman Wood-Fired Glassworking Furnaces: Waste Products and Their Formation Processes.. Journal of Glass Studies.

[46] Degryse P, Freestone I, Schneider J, Jennings S, Drauschke J, Keller D (2010). Technology and provenance of Levantine plant ash glass using Sr-Nd isotope analysis.. Glass in Byzantium - Production, Usage, Analyses RGZM - Tagungen 8.

[47] Andreescu-Treadgold I, Henderson J (2006). Glass from the Mosaics on the West Wall of Torcello's Basilica.. Arte Medievale.

[48] Arletti R, Fiori C, Vandini M (2010). A Study of Glass Tesserae from Mosaics in the Monasteries of Daphni and Hosios Loukas (Greece).. Archaeometry.

[49] Sayre EV, Smith RW (1961). Compositional Categories of Ancient Glass.. Science.

[50] Lankton JW, Dussubieux L (2006). Early glass in Asian maritime trade: A review and an interpretation of compositional analyses.. Journal of Glass Studies.

[51] Boulogne S, Henderson J (2009). Indian Glass in the Middle East? Medieval and Ottoman Glass Bangles from Central Jordan.. Journal of Glass Studies.

[52] Helvaci C, Alonso RN (2000). Borate deposits of Turkey and Argentina; a summary and geological comparison.. Turkish Journal of Earth Science.

[53] Helvaci C, Mordogan H, Colak M, Gundogan I (2004). Presence and distribution of lithium in borate deposits and some recent lake waters of west-central Turkey.. International Geology Review.

[54] Brill RH (1968). The scientific investigation of ancient glasses..

[55] Camacho-Cristobal JJ, Rexach J, Gonzalez-Fontes A (2008). Boron in plants: Deficiency and toxicity.. Journal of Integrative Plant Biology.

[56] Miwa K, Takano J, Omori H, Seki M, Shinozaki K (2007). Plants tolerant of high boron levels.. Science.

[57] Arletti R, Giordani N, Rarpini R, Vezzalini G (2005). Archaeometrical analysis of glass of Western Emilia Romagna (Italy) from the imperial age..

[58] Mirti P, Casoli A, Appolonia L (1993). Scientific Analysis of Roman Glass from Augusta-Praetoria.. Archaeometry.

[59] Silvestri A (2008). The coloured glass of Iulia Felix.. Journal of Archaeological Science.

[60] Silvestri A, Molin G, Salviulo G (2005). Roman and medieval glass from the Italian area: Bulk characterization and relationships with production technologies.. Archaeometry.

[61] Silvestri A, Molin G, Salviulo G (2008). The colourless glass of Iulia Felix.. Journal of Archaeological Science.

