# Supplementary material for: Late Byzantine Mineral Soda High Alumina Glasses from Asia Minor: A New Primary Glass Production Group
Source: PLoS One. 2011 Apr 19;6(4):e18970. doi: 10.1371/journal.pone.0018970 (PMC3079742; doi:10.1371/journal.pone.0018970)
Supplement: Table S1 — EPMA results for Corning glass standards (in wt% of oxides) to monitor the accuracy of the measurements. Absolute deviations (measured – expected value, in wt%) and relative deviations (absolute deviation divided by the expected value, in %) are indicated. (DOC) [file pone.0018970.s001.doc]

|  | **SiO_2_** | **Na_2_O** | **K_2_O** | **CaO** | **MgO** | **Al_2_O_3_** | **FeO** | **TiO_2_** | **MnO** | **P_2_O_5_** | **Cl** | **SO_3_** | **CuO** | **Sb_2_O_5_** | **PbO** |
| --- | --- | --- | --- | --- | --- | --- | --- | --- | --- | --- | --- | --- | --- | --- | --- |
| **Corning A measured** | 66.79 | 14.34 | 2.89 | 5.30 | 2.59 | 0.90 | 1.03 | 0.85 | 1.08 | 0.12 | 0.09 | 0.12 | 1.27 | 1.97 | 0.08 |
| **Corning A expected** | 66.56 | 14.30 | 2.87 | 5.03 | 2.66 | 1.00 | 1.09 | 0.79 | 1.00 | 0.13 |  |  | 1.17 | 1.75 | 0.13 |
| **absolute deviation** | -0.23 | -0.04 | -0.02 | -0.27 | 0.07 | 0.10 | 0.06 | -0.06 | -0.08 | 0.01 | -0.09 | -0.12 | -0.10 | -0.22 | 0.05 |
| **relative deviation** | -0.35 | -0.25 | -0.85 | -5.41 | 2.79 | 9.52 | 5.19 | -7.24 | -7.51 | 11.33 |  |  | -8.94 | -12.86 | 36.44 |
| **Corning B measured** | 61.77 | 17.12 | 1.04 | 9.42 | 1.01 | 4.11 | 0.35 | 0.12 | 0.29 | 0.88 | 0.19 | 0.46 | 2.91 | 0.49 | 0.54 |
| **Corning B expected** | 61.55 | 17.00 | 1.00 | 8.56 | 1.03 | 4.36 | 0.34 | 0.09 | 0.25 | 0.82 | 0.20 | 0.54 | 2.66 | 0.46 | 0.61 |
| **absolute deviation** | -0.22 | -0.12 | -0.04 | -0.86 | 0.02 | 0.25 | -0.01 | -0.03 | -0.04 | -0.06 | 0.01 | 0.08 | -0.25 | -0.03 | 0.07 |
| **relative deviation** | -0.36 | -0.70 | -4.39 | -10.05 | 1.79 | 5.75 | -3.96 | -33.02 | -16.41 | -7.02 | 7.35 | 15.01 | -9.28 | -7.07 | 10.69 |
